# Prognostic impact of corticosteroid maintenance dose and re-escalation in patients with cardiac sarcoidosis

**DOI:** 10.1136/openhrt-2026-004048

**Published:** 2026-03-06

**Authors:** Takuya Nishimura, Kohei Ishibashi, Koshiro Kanaoka, Kenzaburo Nakajima, Takashi Ikee, Daiki Syako, Toshihiro Nakamura, Satoshi Oka, Akinori Wakamiya, Nobuhiko Ueda, Tsukasa Kamakura, Mitsuru Wada, Yuko Inoue, Koji Miyamoto, Takeshi Aiba, Kengo Kusano

**Affiliations:** 1 National Cerebral and Cardiovascular Center, Suita, Japan; 2 Department of Cardiology and Geriatrics, Kochi Medical School Kochi University, Nankoku, Kochi, Japan; 3 Department of Medical and Health Information Management, National Cerebral and Cardiovascular Center, Suita, Japan

**Keywords:** Cardiomyopathies, HEART FAILURE, Inflammation

## Abstract

**Background:**

Japanese guidelines recommend a corticosteroid maintenance dose of 5–10 mg/day for cardiac sarcoidosis (CS); however, the optimal dose remains unclear. This study aimed to evaluate the impact of maintenance dose and corticosteroid re-escalation on prognosis in patients with CS.

**Methods:**

This multicentre retrospective cohort study used data from a Japanese nationwide CS registry. A total of 352 patients diagnosed according to the Japanese Circulation Society 2016 guideline and treated with oral corticosteroids were included. Patients were grouped by maintenance dose: low (<5.0 mg/day), recommended (5.0–10.0 mg/day) and high (>10.0 mg/day). Clinical outcomes were analysed. The main outcome was all-cause mortality.

**Results:**

The low-dose, recommended-dose and high-dose groups comprised 11% (n=40), 78% (n=276) and 10% (n=36) of patients, with mean maintenance doses of 2.2 mg/day, 6.7 mg/day and 16.2 mg/day, respectively. During a median follow-up of 5.12 years, 39 patients (11%) died. Kaplan-Meier survival analysis showed statistically better survival in the recommended dose group, with the high-dose group showing statistically significantly worse outcomes (log-rank p=0.012). Corticosteroid re-escalation occurred in 19% of patients (9% before and 11% after achieving maintenance dose). All-cause mortality was 8% in the recommended-dose group versus 25% in the low-dose and 17% in the high-dose groups. In univariable analyses, re-escalation after achieving maintenance was associated with mortality in the high-dose group (HR 4.34, 95% CI 1.24 to 98.3), whereas re-escalation before achieving maintenance was associated with mortality in the low-dose group (HR 19.41, 95% CI 2.71 to 138.5).

**Conclusions:**

A recommended maintenance dose of corticosteroids was associated with better prognosis in patients with CS. Achieving and maintaining this dose appears critically important in clinical management.

**WHAT IS ALREADY KNOWN ON THIS TOPIC**

- Japanese guidelines recommend a corticosteroid maintenance dose of 5.0–10.0 mg/day for cardiac sarcoidosis, but the prognostic impact of dose selection and the clinical significance of corticosteroid re-escalation have remained uncertain.

**WHAT THIS STUDY ADDS**

- In a multicentre retrospective cohort of 352 patients initiating oral corticosteroids, the recommended-dose group showed the lowest all-cause mortality (8%) compared with the low-dose (25%) and high-dose (17%) groups. Re-escalation after achieving maintenance was associated with higher mortality in the high-dose group (HR 4.34, 95% CI 1.24 to 98.3), whereas re-escalation before achieving maintenance was associated with higher mortality in the low-dose group (HR 19.41, 95% CI 2.71 to 138.5).

**HOW THIS STUDY MIGHT AFFECT RESEARCH, PRACTICE OR POLICY**

- These findings support guideline-recommended maintenance dosing and suggest that the need for re-escalation may flag higher-risk patients who warrant closer monitoring and treatment review, while prospective studies are needed to confirm causality and optimise tapering strategies.

## Introduction

 Sarcoidosis is a systemic granulomatous disease of unknown aetiology. Sarcoid granulomas caused by inflammation can occur in multiple organs, including the heart, and the prognosis of patients with sarcoidosis has been well demonstrated to depend significantly on cardiac involvement.^1^ The involvement of the heart, known as cardiac sarcoidosis (CS), causes heart failure, ventricular arrhythmias and even sudden cardiac death.^2^ Immunosuppressive drugs represent the first-line treatment^3 4^ and non-pharmacological therapies, including pacemaker implantation, implantable cardioverter-defibrillator, cardiac resynchronisation therapy and radiofrequency catheter ablation, are sometimes required for managing CS. Corticosteroids are the cornerstone of immunosuppressive therapy in CS, with methotrexate and infliximab commonly used as second-line agents. The initial corticosteroid dosage typically involves 30 mg/day (0.5 mg/kg/day) or 60 mg/day every other day (1.0 mg/kg/every other day) for 4 weeks. Tapering follows at a rate of 5 mg/day or 10 mg every other day every 2–4 weeks. Current guidelines recommend a maintenance dose of 5–10 mg/day or 10–20 mg/day every other day,^5^ and registry data report a mean maintenance dose of 7.5 mg/day.^6^ However, robust evidence remains lacking regarding the optimal initial dose, tapering strategy and maintenance regimen for corticosteroids in CS.^7^ This study aimed to investigate the impact of maintenance dose and corticosteroid re-escalation on prognosis in patients with CS.

## Methods

### Study population

This study was a multicentre, retrospective cohort study based on a nationwide Japanese survey on CS conducted between 2014 and 2016. Details of the nationwide cohort study components, including data on diagnosis, treatment and clinical outcomes of CS, have been previously published.^6^ A total of 757 patients diagnosed with CS at each hospital were enrolled in this study. The exclusion criteria included: (1) patients who did not meet the diagnostic criteria for CS based on the Japanese Circulation Society (JCS) 2016 guideline (n=219),^8^ (2) patients with insufficient clinical data or those lost to follow-up (n=116), (3) patients who received heart transplants (n=2) and (4) patients who did not receive oral corticosteroid therapy (n=68). Data from 352 patients with CS were ultimately analysed. Atrioventricular block (AVB) was defined as second-degree Mobitz type I and Mobitz type II, advanced or complete AVB. This study was approved by the Institutional Review Committee of the National Cerebral and Cardiovascular Centre (M26-016-5, 4 June 2014) and each participating hospital. Anonymised data were analysed after patients agreed to treatment, and informed consent was obtained using an opt-out approach via posters or leaflets approved by each institutional review committee. The study complied with the principles of the Declaration of Helsinki.

### Patient and public involvement

Patients and the public were not involved in the design, conduct, reporting or dissemination plans of this study.

### Corticosteroid therapy and re-escalation

No clear standards existed for the initial or maintenance dose of corticosteroids; therefore, attending physicians determined these doses at their discretion. In this study, the maintenance dose of corticosteroids was defined as the dose that remained unchanged for at least 1 year. The re-escalation of corticosteroids was defined as an increase in dose during follow-up. The analysis of re-escalation included both the presence or absence of re-escalation and the timing of re-escalation, categorised as occurring before or after achieving the maintenance dose.

### Study protocol

**Figure 1:**
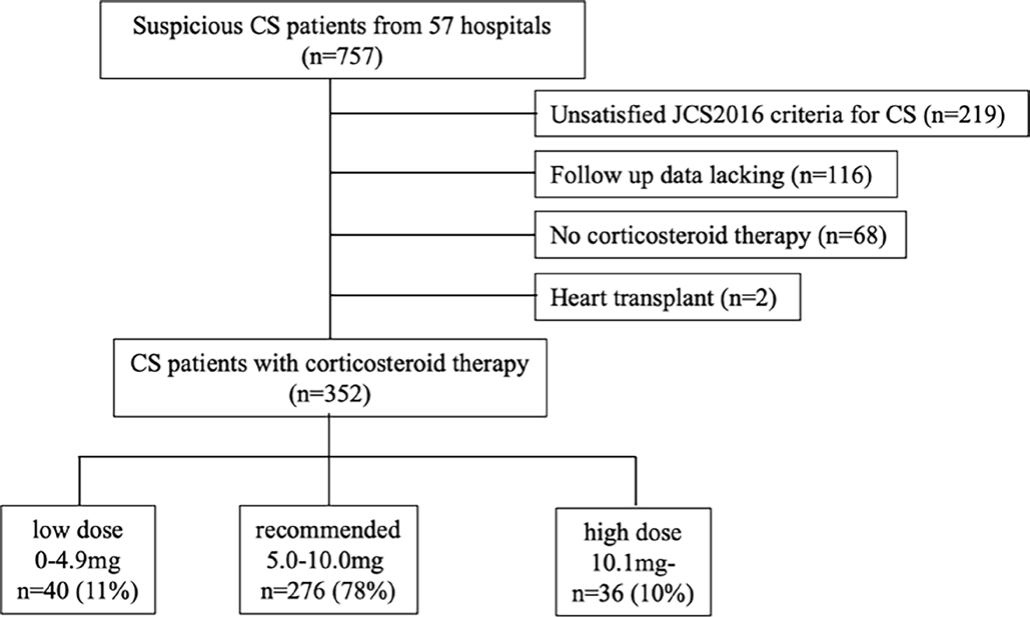
Flow chart illustrating the inclusion and exclusion criteria for cardiac sarcoidosis (CS) based on the Japanese Circulation Society (JCS) guidelines.

This study included 352 patients who were introduced to oral corticosteroids (figure 1). The median follow-up period was 5.12 (IQR 2.46–8.62) years. Patients were divided into two groups based on maintenance dose: the recommended dose group (5.0–10.0 mg) and the non-recommended dose group (0–4.9 mg or ≥10.1 mg), in accordance with current guideline recommendations. The non-recommended dose group was further subdivided into the low-dose (0–4.9 mg) and high-dose (≥10.1 mg) groups. Clinical characteristics at the time of CS diagnosis were evaluated across these maintenance dose categories. Prognosis was assessed by comparing all-cause mortality and also evaluated with and without corticosteroid re-escalation. Furthermore, prognosis and clinical factors associated with all-cause mortality were analysed separately in the low-dose and high-dose groups.

This study focused on corticosteroid monotherapy and investigated maintenance dosing and re-escalation patterns. These data were collected through physician-completed questionnaires based on a review of each hospital’s medical records.

### Statistical analysis

All statistical analyses were performed using EZR V.1.52 (Saitama Medical Centre, Jichi Medical University, Saitama, Japan), a graphical user interface for R V.4.02 (The R Foundation for Statistical Computing, Vienna, Austria). This software represents a modified version of R commander, designed to incorporate statistical functions frequently used in biostatistics.^9^ Continuous variables were expressed as the mean±SD for variables with normal distribution and as median and IQR for variables with non-normal distribution. Categorical data were expressed as numbers and percentages. Group differences were analysed using statistical methods appropriate to the type and distribution of the data. For continuous variables, one-way analysis of variance (ANOVA) was applied to normally distributed data, whereas the Kruskal-Wallis test was used for non-normally distributed data. For categorical variables, either the χ^2^ or Fisher’s exact test was used. A p value <0.05 in the χ^2^ and Fisher’s exact tests was considered statistically significant. When the Kruskal-Wallis test or one-way ANOVA showed a significant difference (p<0.05), post hoc analyses were performed using the Bonferroni method; p values<0.017 were considered statistically significant for multiple comparisons. Kaplan-Meier curves and log-rank tests were employed to compare all-cause mortality between groups according to maintenance dose and re-escalation status. Survival time was defined as the duration from CS diagnosis to the occurrence of all-cause mortality and was used as the outcome variable in these analyses. A univariate Cox proportional hazards analysis was conducted to identify variables significantly associated with all-cause mortality in patients with CS. The Cox proportional model results were presented as HRs, p values and 95% CIs. The multivariable Cox proportional hazards analysis for all-cause mortality included age, sex, left ventricular ejection fraction (LVEF) with a p value <0.10 in the univariable analysis and corticosteroid maintenance dose group as covariables. Multicollinearity was assessed using variance inflation factors (VIFs), and multicollinearity was considered absent when all VIF values were <2.0. The proportional hazards assumption was assessed using Schoenfeld residuals. In addition, to explore potential time-varying associations, we performed time-stratified Cox regression analyses by dividing follow-up at 5 years, approximately corresponding to the median follow-up duration. Statistical significance was set at p<0.05. Infection-related death rates were calculated as the number of events divided by the total person-years of follow-up and were expressed as events per 100 person-years. 95% CIs for these rates were estimated using the Poisson distribution.

## Results

### Patient characteristics at baseline

The proportions of patients in the low-dose, recommended-dose and high-dose groups were 11% (n=40), 78% (n=276), and 10% (n=36), respectively. Among the 352 patients enrolled, 323 were treated with corticosteroid monotherapy, whereas 29 received concomitant immunosuppressive therapy. Additional immunosuppressants were used in 5.0%, 8.3% and 11.1% of the low-dose, recommended-dose and high-dose groups, respectively. The baseline clinical characteristics are summarised in table 1. The mean age was 59.4 years, and women accounted for 71% (n=250). Systemic CS and isolated CS accounted for 92% (n=325) and 8% (n=27), respectively. Clinical and histological diagnoses accounted for 82% (n=289) and 18% (n=63), respectively. At the time of diagnosis, the initial cardiac arrhythmias included AVB in 40%, non-sustained ventricular tachycardia in 13%, and ventricular tachycardia (VT) in 17% of patients. The mean LVEF was 51%, and late gadolinium enhancement (LGE) on cardiac MRI (CMRI) was observed in 85% (161/188). No significant differences in baseline patient characteristics were observed among three groups.

**Table 1:** Baseline characteristics of the study population

	All patients (n=352)	Low-dose (0–4.9 mg) (n=40)	Recommended-dose (5.0–10.0 mg) (n=276)	High-dose (>10.1 mg) (n=36)	P value
Corticosteroid maintenance dose (mg)	7.2±4.0	2.2±1.2	6.7±2.1	16.2±3.6	
Age (years)	59.4±10.8	58.6±9.4	59.9±10.5	56.9±13.6	0.279
Sex; female	250 (71)	28 (70)	202 (73)	20 (55)	0.093
Diagnosis					
Systemic CS Isolated CS Clinical Histological	325 (92) 27 (8) 289 (82) 63 (18)	35 (87) 5 (13) 33 (82) 7 (18)	258 (93) 18 (7) 223 (80) 53 (20)	32 (89) 4 (11) 33 (92) 3 (8)	0.296 0.277
Extra-cardiac involvement					
Lung Eye Skin	243 (69) 81 (23) 64 (18)	24 (60) 8 (20) 6 (15)	195 (70) 63 (23) 53 (19)	24 (67) 10 (28) 5 (14)	0.375 0.729 0.731
Medication					
Beta-blocker ACE-i/ARB Sodium channel blocker Potassium channel blocker	249 (70) 197 (56) 22 (6) 110 (31)	28 (70) 24 (60) 2 (5) 15 (37)	191 (69) 152 (55) 18 (6) 85 (30)	30 (83) 21 (58) 2 (5) 10 (28)	0.214 0.804 0.918 0.619
Arrhythmia					
AVB NSVT VF/VT	143 (40) 46 (13) 61 (17)	15 (38) 5 (12) 7 (17)	117 (42) 32 (11) 46 (17)	11 (30) 9 (25) 8 (22)	0.370 0.093 0.676
BNP, (ng/L)	160(55–431)	108(32–474)	165(56–439)	177(62–365)	0.561
LVEF, (%)	51(37–62)	52(36–64)	51(37–62)	44(39–60)	0.679
LVSWT	161 (46)	20 (50)	129 (46)	12 (33)	0.266
^67^Ga scintigraphy uptake	238/337 (70)	28/37 (75)	188/265 (70)	22/35 (63)	0.479
SPECT perfusion defect present	189/216 (87)	15/19 (79)	154/176 (87)	20/21 (95)	0.315
CMRI myocardial LGE present	161/188 (85)	12/16 (75)	130/153 (85)	19/19 (100)	0.055
Device implantation					
Pacemaker/CRT-P ICD/CRT-D	141 (40) 122 (34)	14 (35) 5 (12)	116 (42) 104 (37)	11 (30) 13 (36)	0.340 0.940

Values are presented as median (IQR), mean±SD, number (%) or number of positive findings per number of studied patients (%).

ACE-i, ACE inhibitor; ARB, angiotensin II receptor blocker; AVB, atrioventricular block; BNP, brain natriuretic peptide; CMRI, cardiac MRI; CRT-D, cardiac resynchronization therapy-defibrillator; CRT-P, cardiac resynchronization therapy-pacing; CS, cardiac sarcoidosis; 67Ga, gallium-67; ICD, implantable cardioverter defibrillator; IQR, interquartile range; LGE, late gadolinium enhancement; LVEF, left ventricular ejection fraction; LVSWT, left ventricular septal wall thinning; NSVT, non-sustained ventricular tachycardia; SPECT, single photon emission computed tomography; VF, ventricular fibrillation; VT, ventricular tachycardia.

### Survival analysis during three corticosteroid maintenance dose groups

The median follow-up period was 5.12 years. During the follow-up period, 39 all-cause mortality events occurred (table 2). The causes of death were as follows: heart failure (n=14), sudden cardiac death (n=10), infection (n=9), stroke (n=3) and malignancy (n=3).

**Table 2:** All-cause mortality and corticosteroid re-escalation according to maintenance dose

	All patients (n=352)	Low-dose (0–4.9 mg) (n=40)	Recommended-dose (5.0–10.0 mg) (n=276)	High-dose (>10.1 mg) (n=36)	P value
Follow-up period, years	5.12 (2.65–8.62)	7.09 (3.35–10.72)	5.15 (2.50–8.62)	2.57 (0.88–5.78)	
All-cause mortality	39 (11)	10 (25)*	23 (8)*	6 (17)*	0.012
Re-escalation					
Re-escalation before maintenance dose	31 (9)	2 (5)	22 (8)	7 (19)	0.068
Re-escalation after maintenance dose	38 (11)	8 (30)	25 (9)	5 (14)	0.082

Values are presented as median (IQR), mean±SD deviation or number (%).

* Indicates that the post hoc analysis using the Bonferroni method revealed a significant difference between two groups (p<0.017).

**Figure 2:**
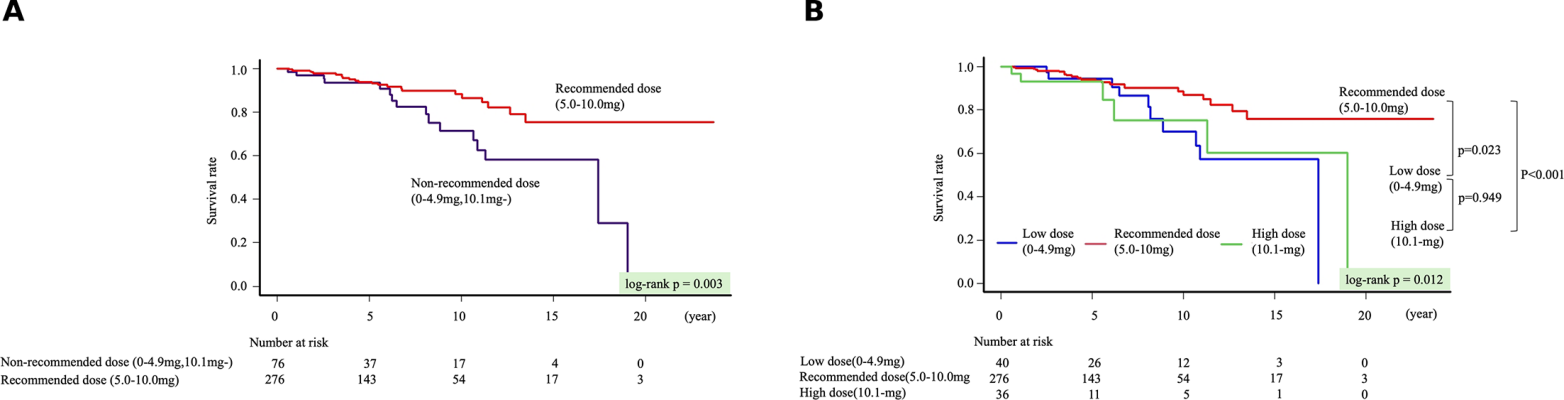
(A) Compares all-cause mortality among the recommended and non-recommended dose groups (low-dose and high-dose). (**B**) Compares all-cause mortality among the recommended-dose, low-dose and high-dose groups.

**Figure 3:**
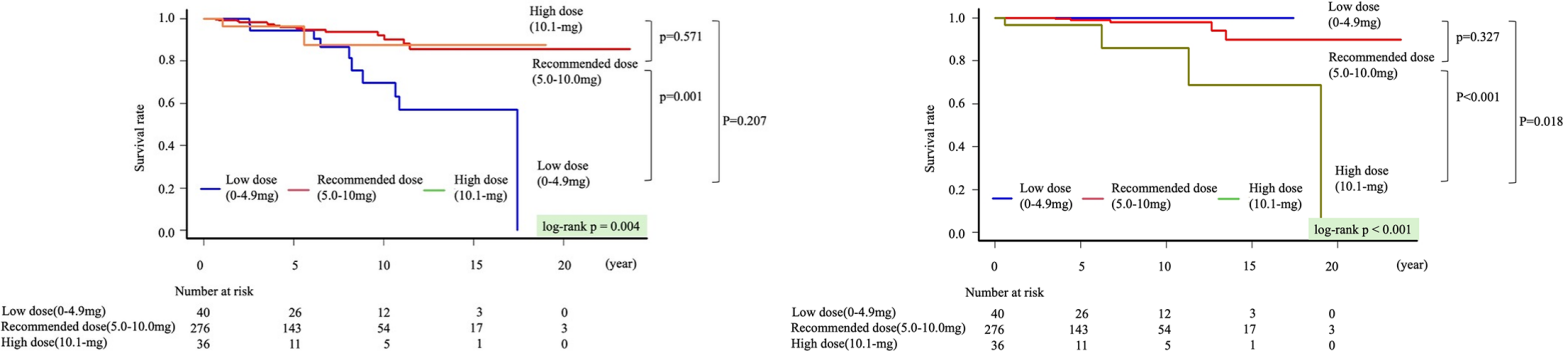
(A) Compares cardiovascular death among the recommended-dose, low-dose and high-dose groups. (**B**) Compares infection death among the recommended-dose, low-dose and high-dose groups.

Kaplan-Meier curves for all-cause mortality showed a statistically significantly poorer prognosis in the non-recommended dose group compared with the recommended dose group (p=0.003) (figure 2A). Notably, the prognosis in the high-dose group was significantly poorer than in the recommended dose group (log-rank p<0.001) (figure 2B). Kaplan-Meier analysis for all-cause mortality performed in the 323 patients after excluding the 29 patients who received additional immunosuppressive agents demonstrated that the recommended-dose group continued to exhibit the most favourable prognosis (p=0.016) (online supplemental figure 1). In the multivariable Cox proportional hazards model, age (HR 1.05, 95% CI 1.01 to 1.09, p=0.005) and LVEF (HR 0.97, 95% CI 0.95 to 0.99, p=0.002) were independently statistically significantly associated with all-cause mortality. Compared with the recommended-dose group, the low-dose group showed a significantly higher mortality risk (HR 2.68, 95% CI 1.25 to 5.76, p=0.011), as did the high-dose group (HR 3.01, 95% CI 1.10 to 8.26, p=0.031) (table 3). The proportional hazards assumption was evaluated using Schoenfeld residuals. The global test did not demonstrate a statistically significant violation of the proportional hazard assumption (GLOBAL p=0.268), and no statistically significant time dependency was observed for the corticosteroid maintenance dose groups (p=0.349). To explore potential time-varying associations, we conducted time-stratified Cox regression analyses by dividing follow-up at 5 years. 16 deaths occurred within the first 5 years and 23 beyond 5 years. During the first 5 years, maintenance dose was not statistically significantly associated with mortality (low dose: HR 1.27, 95% CI 0.28 to 5.78; p=0.754; high dose: HR 2.39, 95% CI 0.52 to 10.99; p=0.261). Beyond 5 years, both the low-dose and high-dose groups were statistically significantly associated with increased mortality compared with the recommended-dose group (low dose: HR 3.98, 95% CI 1.55 to 10.17; p=0.004; high dose: HR 4.33, 95% CI 1.27 to 14.79; p=0.019). In addition, all VIF values were below 2.0, indicating that no multicollinearity was present among the variables in the model. According to a composite of cardiovascular death or VT/ventricular fibrillation (VT/VF), the event counts were 10/40 (25%) in the low-dose group, 41/276 (15%) in the recommended-dose group and 5/36 (14%) in the high-dose group. Kaplan-Meier curves showed no significant difference among three groups (p=0.120). In addition, we separately evaluated cardiovascular death and VT/VF events. Cardiovascular (CV) death differed significantly among three groups (p=0.004) (figure 3A), whereas VT/VF events did not show a significant difference (p=0.141). Infection-related deaths occurred in 0, five and four patients, in the low-dose, recommended-dose and high-dose groups, respectively. The corresponding infection-related death rates were 0.00, 0.29 and 2.68 per 100 person-years, respectively (95% CIs 0.00 to 1.25, 0.10 to 0.69 and 0.73 to 6.87). The incidence of infection death differed significantly among three groups (p<0.001) (Figure 3B).

**Table 3:** Prognostic factors for all-cause mortality in all population

	Univariable			Multivariable		
	HR	P value	95% CI	HR	P value	95% CI
Age (1-year increase)	1.04	0.023	1.00 to 1.07	1.05	0.005	1.01 to 1.09
Female sex (vs male sex)	0.83	0.589	0.41 to 1.64	0.73	0.415	0.35 to 1.53
Histological CS (vs clinical CS)	0.79	0.528	0.38 to 1.63			
iCS (vs systemic CS)	1.56	0.358	0.60 to 4.00			
AVB at CS diagnosis	0.59	0.127	0.30 to 1.16			
VT at CS diagnosis	0.68	0.387	0.28 to 1.62			
LVEF (1% increase)	0.97	0.002	0.95 to 0.99	0.97	0.002	0.95 to 0.99
Abnormal uptake of ^67^Ga scintigraphy or ^18^F-FDG PET in the heart	3.31	0.266	0.40 to 27.30			
Corticosteroid re-escalation	0.87	0.700	0.41 to 1.80			
Corticosteroid maintenance dose						
Recommended-dose Low-dose High-dose	Reference 2.36 2.82	0.024 0.025	1.11 to 4.97 1.14 to 7.00	Reference 2.68 3.01	0.011 0.031	1.25 to 5.76 1.10 to 8.26

AVB, atrioventricular block; CS, cardiac sarcoidosis; 18F-FDG PET, 18F-fluorodeoxyglucose positron emission tomography; 67Ga, gallium-67; iCS, isolated cardiac sarcoidosis; LVEF, left ventricular ejection fraction; VT, ventricular tachycardia.

### Corticosteroid re-escalation and prognosis

Corticosteroid re-escalation occurred in 19% (n=67) of patients. The baseline clinical characteristics with and without re-escalation are summarised in online supplemental table 1. Patients in the re-escalation group tended to be younger and exhibited a higher proportion of isolated CS. Re-escalation occurred in 9% (n=31) before and 11% (n=38) after achieving the maintenance dose (Table 2). Although a significant difference in re-escalation was observed among three groups (p=0.031), no significant difference was noted in the timing of re-escalation (before vs after maintenance dose) among the groups. The Kaplan-Meier analysis comparing patients with and without re-escalation demonstrated no significant difference in all-cause mortality (p=0.115).

### Corticosteroid re-escalation and prognosis in the non-recommended dose group

**Figure 4:**
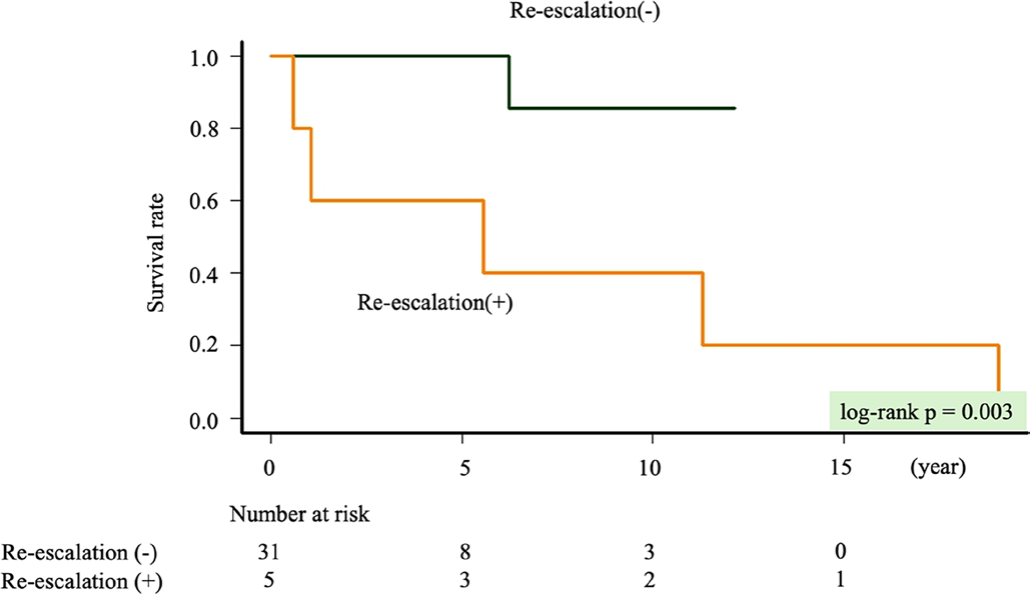
Compares all-cause mortality between patients with and without re-escalation after achieving the maintenance dose in the high-dose group.

In the non-recommended dose group, Kaplan-Meier curves revealed no significant difference in all-cause mortality between those with and without re-escalation (p=0.364). In the high-dose group, re-escalation after achieving the maintenance dose was statistically significantly associated with higher all-cause mortality in univariable analysis (p=0.003) (figure 4). In univariable analyses, age, LVEF and re-escalation after achieving the maintenance dose were statistically significant for all-cause mortality in the high-dose group (table 4). In the low-dose group, re-escalation before achieving the maintenance dose was statistically significant for all-cause mortality (table 5).

**Table 4:** Prognostic factors for all-cause mortality in the high-dose group (>10.0 mg)

	Univariable		
	HR	P value	95% CI
Age (1-year increase)	1.04	0.037	1.00 to 1.07
Female sex (vs male sex)	2.70	0.172	0.63 to 11.55
Histological CS (vs clinical CS)	0.27	0.165	0.06 to 1.91
iCS (vs systemic CS)	0.00	0.999	0 to Infinity
AVB at CS diagnosis	0.41	0.209	0.08 to 1.66
VT at CS diagnosis	0.65	0.618	0.15 to 4.48
LVEF (1% increase)	0.91	0.001	0.85 to 0.97
Abnormal uptake of ^67^Ga scintigraphy or ^18^F-FDG PET in the heart	2.08	0.339	0.42 to 8.54
Corticosteroid re-escalation Re-escalation before maintenance dose Re-escalation after maintenance dose	0.44 4.34	0.457 0.005	0.05 to 3.72 1.24 to 98.3

AVB, atrioventricular block; CS, cardiac sarcoidosis; 18F-FDG PET, 18F-fluorodeoxyglucose positron emission tomography; 67Ga, gallium-67; iCS, isolated cardiac sarcoidosis; LVEF, left ventricular ejection fraction; VT, ventricular tachycardia.

**Table 5:** Prognostic factors for all-cause mortality in the low-dose group (0–4.9 mg)

	Univariable		
	HR	P value	95% CI
Age (1-year increase)	0.97	0.462	0.91 to 1.03
Female sex (vs male sex)	0.48	0.314	0.11 to 1.99
Histological CS (vs clinical CS)	2.50	0.389	0.30 to 20.2
iCS (vs systemic CS)	0.74	0.786	0.09 to 6.02
AVB at CS diagnosis	1.21	0.776	0.32 to 4.51
VT at CS diagnosis	2.61	0.180	0.63 to 10.7
LVEF (1% increase)	0.98	0.436	0.94 to 1.02
Abnormal uptake of ^67^Ga scintigraphy or ^18^F-FDG PET in the heart	3.31	0.266	0.40 to 27.3
Corticosteroid re-escalation Re-escalation before maintenance dose Re-escalation after maintenance dose	19.41 0.23	0.003 0.174	2.71 to 138.5 0.02 to 1.90

AVB, atrioventricular block; CS, cardiac sarcoidosis; 18F-FDG PET, 18F-fluorodeoxyglucose positron emission tomography; 67Ga, gallium-67; iCS, isolated cardiac sarcoidosis; LVEF, left ventricular ejection fraction; VT, ventricular tachycardia.

### Prognostic impact of extracardiac involvement

Kaplan-Meier curves revealed no significant difference in all-cause mortality between systemic CS and isolated CS (p=0.354) (online supplemental figure 2). In the multivariable Cox proportional hazards model, extracardiac involvement was not independently associated with mortality (HR 1.38, 95% CI 0.49 to 3.91, p=0.542) (online supplemental table 2).

## Discussion

This study yielded the following major findings: (1) the non-recommended dose group showed significantly poorer prognosis compared with the recommended dose groups, with the high-dose group showing particularly worse outcomes; (2) corticosteroid re-escalation occurred in 19% of patients, including 9% who underwent re-escalation before achieving the maintenance dose and 11% who underwent re-escalation after achieving the maintenance dose and the frequency of corticosteroid re-escalation before and after achieving the maintenance dose did not significantly differ among the recommended, low-dose and high-dose groups and (3) although corticosteroid re-escalation did not significantly influence prognosis across the entire cohort of patients with CS, re-escalation before achieving the maintenance dose served as a prognostic predictor in the low-dose group, whereas corticosteroid re-escalation after achieving the maintenance dose was a prognostic predictor in the high-dose group. These findings emphasise the clinical importance of achieving and maintaining the recommended corticosteroid dose in managing CS. Furthermore, re-escalation after achieving the maintenance dose in the high-dose group, in which the corticosteroid dose did not fall within the recommended range, may suggest the need to consider additional immunosuppressive therapies beyond corticosteroids.

Sarcoidosis is a multisystemic inflammatory disease characterised by the formation of immune granulomas in various organs, including the heart.^1^ Cardiac involvement occurs in an estimated 5–10% of patients with sarcoidosis. Accurate diagnosis of CS is crucial, as cardiac involvement serves as a prognostic factor in approximately 60–70% of patients with systemic sarcoidosis.^10^,^12^ Immunosuppressive therapy with corticosteroids is generally the first choice for cardiac involvement, and corticosteroid therapy remains the cornerstone of management following a diagnosis of CS.^5 13^ Due to the heterogeneity in disease activity, disease progression and treatment response, standardised protocols for initial corticosteroid dosage, tapering regimens and maintenance dosing have not yet been established.^7^ This study investigated the relationship between corticosteroid maintenance and re-escalation patterns and their impact on prognosis. Regarding the association between corticosteroid maintenance dose and survival outcomes, a higher survival rate was observed in the recommended dose group compared with the non-recommended dose group (low-dose and high-dose groups). These findings appear to support the current guidelines issued in Japan,^5^ which recommend a corticosteroid maintenance dose of 5.0–10.0 mg/day. In the present study, the proportional hazards assumption was not statistically violated according to the global Schoenfeld residual test. However, exploratory time-stratified analyses suggested that the association between maintenance dose and mortality may strengthen over longer follow-up. Specifically, no statistically significant difference was observed within the first 5 years, whereas both the low-dose and high-dose groups were associated with significantly increased mortality beyond 5 years. These findings indicate that the prognostic impact of corticosteroid maintenance dose may become more evident during long-term follow-up. The absence of early differences may partly reflect the dominant influence of baseline disease severity in the initial phase after diagnosis, whereas maintenance strategy may play a more substantial role in determining long-term outcomes. In additional analyses of specific causes of death, CV death was observed more frequently in the low-dose group, whereas infection-related death was more common in the high-dose group. These findings suggest that insufficient suppression of cardiac inflammatory activity may contribute to adverse CV outcomes in patients receiving lower maintenance doses, whereas higher steroid exposure may increase vulnerability to serious infections.

No statistically significant association was found between corticosteroid re-escalation alone and all-cause mortality in patients with CS. However, in univariate analyses, re-escalation before achieving the maintenance dose in the low-dose group was statistically significant (p<0.05) for all-cause mortality. The finding suggests that a corticosteroid maintenance dose of 5 mg or less may not provide sufficient control of disease activity in patients who require dose escalation before achieving maintenance levels. Careful consideration should be given before maintaining corticosteroid therapy at 5 mg or less in these patients. When dose reduction to 5 mg or less becomes necessary due to adverse effects, the addition of other immunosuppressive therapies may be warranted. Previous studies have reported the potential cardiac benefits of methotrexate when administered in combination with low-dose maintenance corticosteroids or during tapering in cases of CS relapse due to poor response or corticosteroid-related adverse effects.^14 15^ Re-escalation after achieving the maintenance dose in the high-dose group also was statistically significant (p<0.05) for all-cause mortality. This finding suggests that disease activity may remain inadequately controlled in patients requiring maintenance doses exceeding 10 mg/day. For such patients, combining corticosteroids with other immunosuppressive agents may improve disease control. Steroid-sparing immunosuppressive therapies represent a viable treatment strategy in patients whose disease remains active despite corticosteroid use or who develop significant corticosteroid-related side effects. These therapies can reduce fluorodeoxyglucose (FDG uptake on imaging and help minimise adverse effects associated with corticosteroids.^16^

However, because the number of all-cause mortality events was small (10 in the low-dose group and six in the high-dose group), it was not possible to perform multivariate analysis. As a result, the statistical significance observed is based solely on univariate analysis, and these factors should not be considered independent predictors. In addition, we evaluated the events among the 29 patients who received concomitant immunosuppressive therapy; however, because the number of cases in both the low-dose and high-dose groups was extremely small, a formal statistical analysis was not performed for this subgroup alone (online supplemental table 3). The diagnostic criteria used in this study warrant consideration when interpreting our findings. Patients were diagnosed according to the JCS 2016 guideline, which differs from other widely used diagnostic criteria, such as the Heart Rhythm Society 2014 consensus statement and the recent American Heart Association 2024 scientific statement.^17^ The JCS criteria may be more sensitive but potentially less specific and thus might have allowed inclusion of patients who would not meet diagnostic criteria in other regions. In particular, isolated CS remains controversial: while the JCS 2016 guideline permits a diagnosis based solely on clinical manifestations and advanced cardiac imaging, several recent studies have demonstrated that genetic cardiomyopathies or other myocardial diseases can mimic isolated CS. Many experts therefore consider genetic testing, pedigree analysis and/or endomyocardial biopsy to be necessary for diagnostic confirmation and to exclude alternative conditions, such as giant cell myocarditis or amyloidosis. The relatively low extent of LGE observed in the low-dose corticosteroid group might indicate milder myocardial involvement, which could increase the risk of diagnostic uncertainty. This potential limitation should be considered, as it may have affected both the assessment of disease severity and the evaluation of treatment outcomes in this subgroup. These diagnostic differences could affect the homogeneity of our study cohort and limit the external validity of our findings. Furthermore, we observed that heart failure was the predominant cause of cardiovascular death in our cohort, whereas arrhythmic deaths have been reported more frequently in some Western series. Such divergence may reflect differences in diagnostic criteria (potentially identifying distinct CS phenotypes or disease stages), genetic predispositions or regional variations in disease presentation and management strategies. These aspects should be considered when comparing our results with those from international cohorts.

### Limitations

This study has several limitations. First, as a retrospective multicentre analysis, detailed information on the reasons for corticosteroid re-escalation could not be obtained. Although a supplementary questionnaire was conducted, information remained incomplete; thus, re-escalation was defined simply as any dose increase during follow-up. In previous reports as well, disease relapse has been defined as the need for treatment intensification, such as an increase in corticosteroid re-escalation.^18^ Consequently, it is difficult to determine whether higher maintenance doses reflect greater disease activity or a direct adverse effect of corticosteroid therapy. Second, the initial corticosteroid dose, tapering strategy and access to follow-up cardiac imaging (eg, 18F-FDG positron emission tomography or CMRI) were determined by each attending physician, leading to potential heterogeneity in management. Third, the JCS 2016 recommendations for maintenance corticosteroid dose (5–10 mg/day) are based on expert consensus rather than randomised evidence and were primarily developed for monotherapy. Because steroid-sparing agents were infrequently and inconsistently used in our cohort, the applicability of our findings to combination therapy remains uncertain. Fourth, body weight data were inconsistently available, precluding reliable weight-adjusted dose analysis, even though corticosteroid pharmacodynamics may vary by body size. Fifth, the lack of information regarding heart failure-related hospitalisations limited our ability to adequately evaluate these outcomes. Finally, selection bias related to the inclusion and exclusion criteria—such as the exclusion of patients with missing data, insufficient follow-up or without corticosteroid therapy—may have reduced external validity and left residual confounding by indication. Accordingly, these findings should be interpreted as hypothesis-generating, as residual confounding by disease severity and treatment indication may have influenced the observed associations. Further prospective studies are warranted to validate these findings and to determine the optimal long-term maintenance strategy.

## Conclusion

Maintaining a corticosteroid dose within the recommended range was associated with improved prognosis in patients with CS.

## Supplementary material

10.1136/openhrt-2026-004048 online supplemental file 1

10.1136/openhrt-2026-004048 online supplemental file 2

10.1136/openhrt-2026-004048 online supplemental file 3

10.1136/openhrt-2026-004048 online supplemental file 4

10.1136/openhrt-2026-004048 online supplemental file 5

10.1136/openhrt-2026-004048 online supplemental file 6

## Data Availability

Data are available upon reasonable request.
