# Supplementary figures and images for: Prognostic impact of corticosteroid maintenance dose and re-escalation in patients with cardiac sarcoidosis
Source: Open Heart. 2026 Mar 6;13(1):e004048. doi: 10.1136/openhrt-2026-004048 (PMC12970080; doi:10.1136/openhrt-2026-004048)

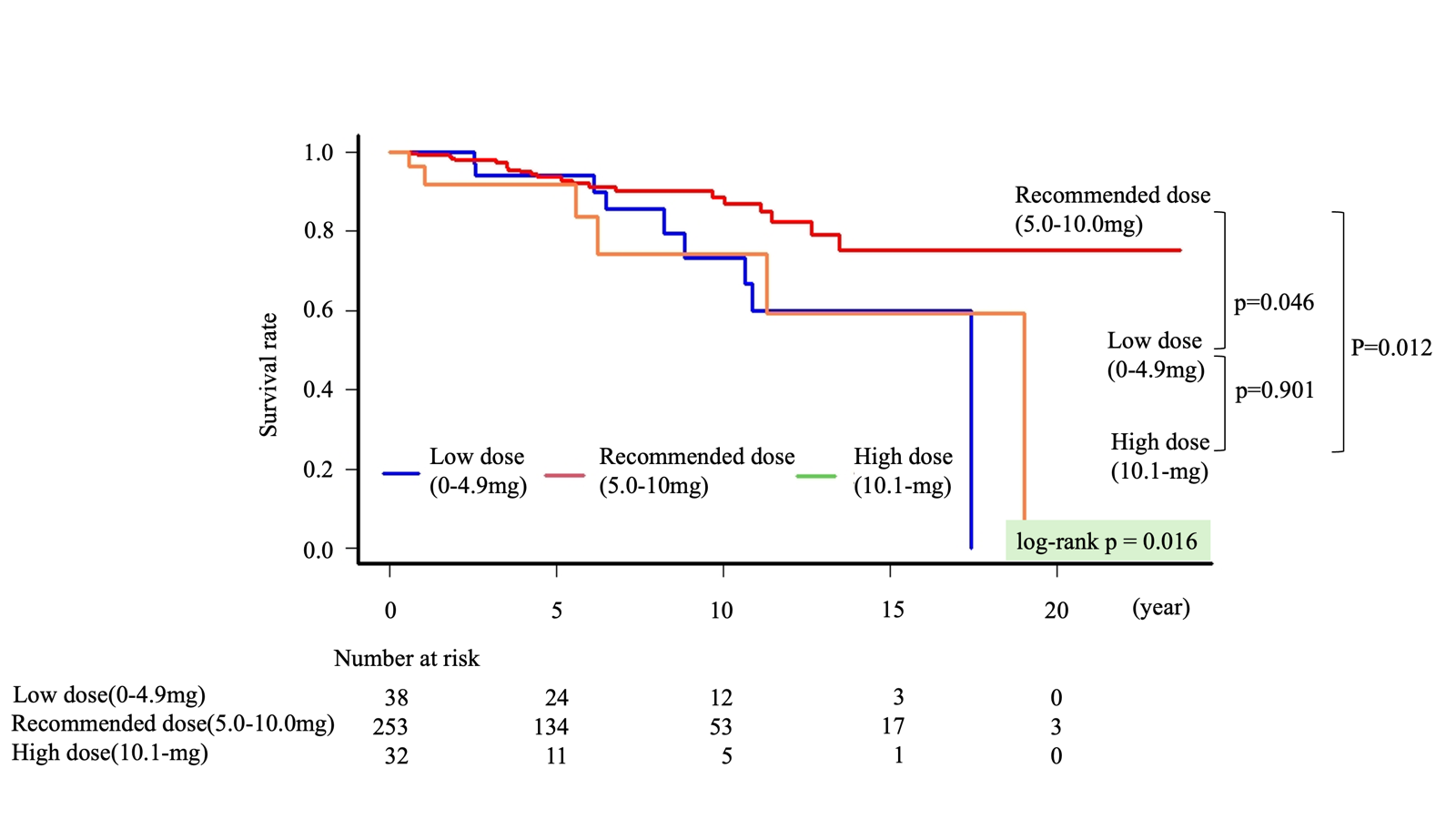

Supplement: online supplemental file 1 [file openhrt-13-1-s001.tif]

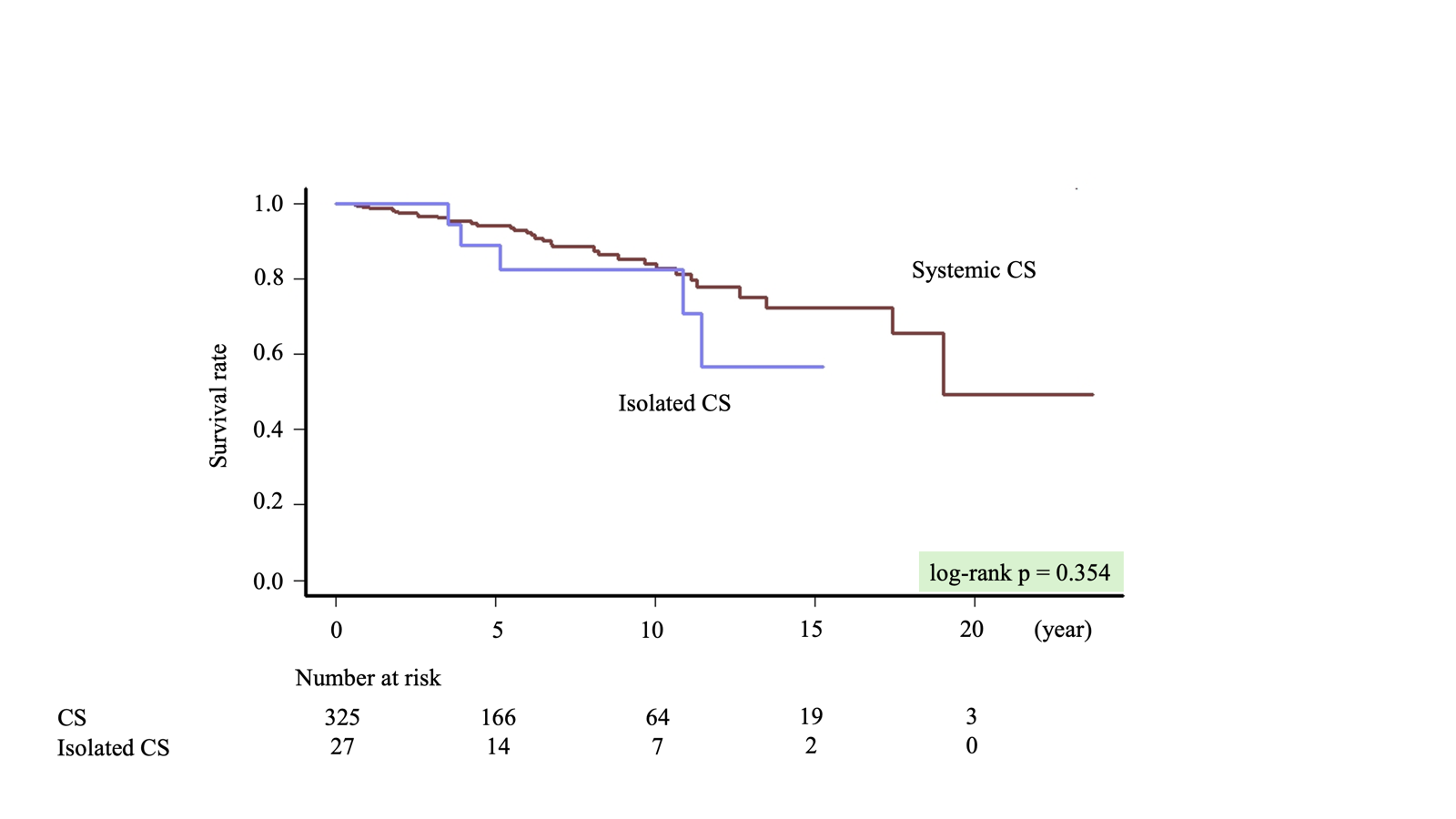

Supplement: online supplemental file 2 [file openhrt-13-1-s002.tif]

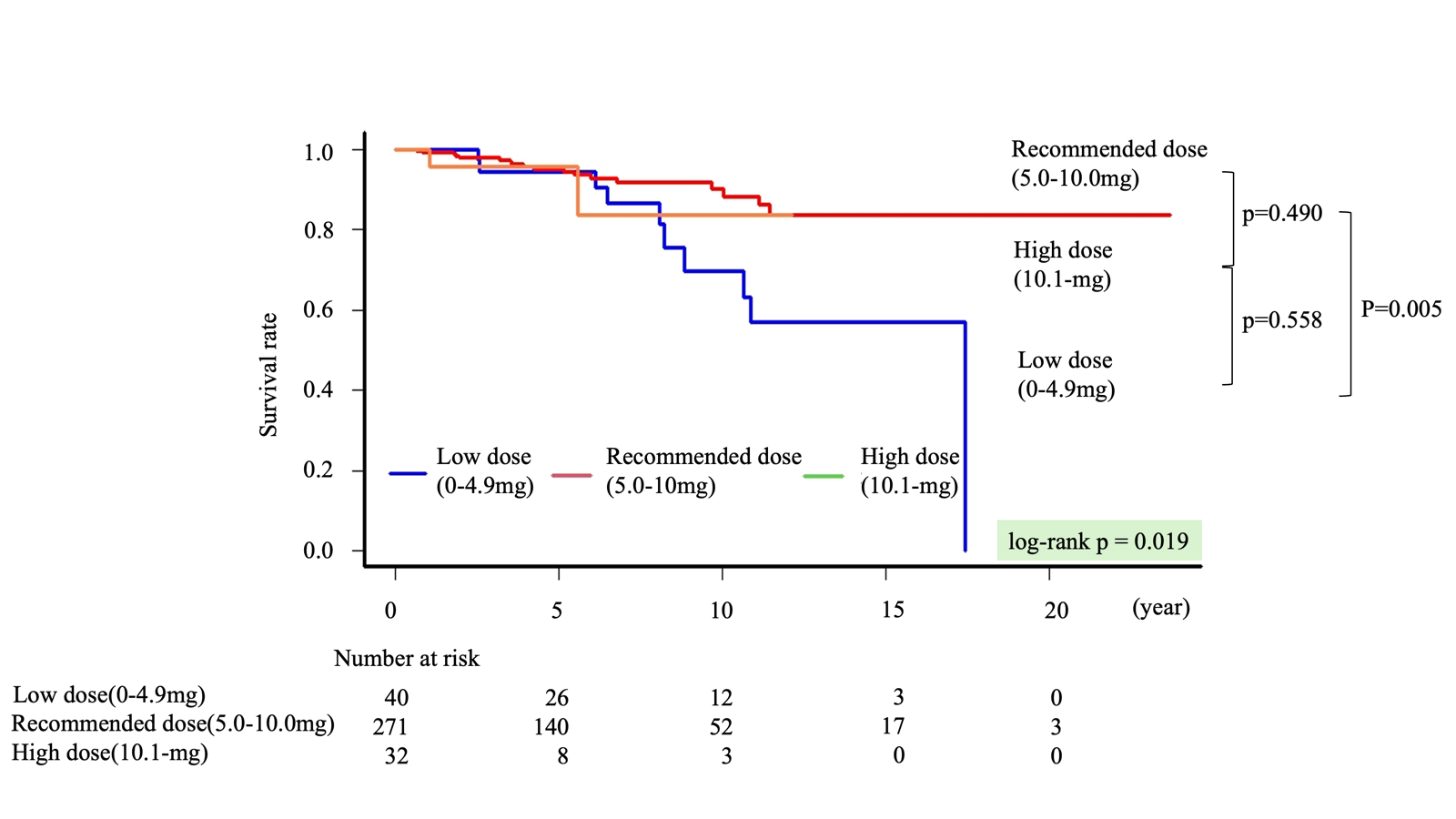

Supplement: online supplemental file 3 [file openhrt-13-1-s003.tif]
