# Supplementary material for: Prognostic impact of corticosteroid maintenance dose and re-escalation in patients with cardiac sarcoidosis
Source: Open Heart. 2026 Mar 6;13(1):e004048. doi: 10.1136/openhrt-2026-004048 (PMC12970080; doi:10.1136/openhrt-2026-004048)
Supplement: online supplemental file 4 [file openhrt-13-1-s004.docx]

Supplemental Table 1 baseline characteristics were compared according to the presence or absence of re-escalation

|  | Re-escalation (+)  (n=67) | Re-escalation (-)  (n=285) | p-value |
| --- | --- | --- | --- |
| Corticosteroid maintenance dose (mg) | 7.36 ± 4.9 | 7.2 ± 3.8 | 0.713 |
| Age (years) | 56.0± 10.6 | 60.2 ± 10.7 | 0.017 |
| Sex; female | 44 (65) | 206 (72) | 0.283 |
| Diagnosis  Systemic CS  Isolated CS  Clinical  Histological | 58 (87)  9 (13)  54 (81)  13 (19) | 267 (94)  18 (6)  235 (83)  50 (17) | 0.086  0.048  0.720  0.720 |
| Extra-cardiac involvement  Lung  Eye  Skin | 45 (67)  14 (21)  9 (13) | 198 (69)  67 (23)  55 (19) | 0.712  0.647  0.262 |
| Medication  Beta-blocker  ACE-i/ARB  Sodium channel blocker  Potassium channel blocker | 54 (80)  36 (54)  8 (12)  26 (39) | 195 (68)  161 (56)  14 (5)  84 (29) | 0.048  0.682  0.155  0.138 |
| Arrhythmia  AVB  NSVT  VF/VT | 23 (34)  8 (12)  14 (21) | 120 (42)  38 (13)  47 (16) | 0.243  0.760  0.391 |
| BNP, (ng/L) | 140 [46–284] | 167 [58–450] | 0.203 |
| LVEF, (%) | 49 [35–60] | 51 [38–63] | 0.300 |
| LVSWT | 35 (52) | 126 (44) | 0.275 |
| ^67^Ga scintigraphy uptake | 41/62 (66) | 197/275 (71) | 0.440 |
| SPECT perfusion defect present | 42/50 (84) | 147/166 (88) | 0.463 |
| CMRI myocardial LGE present | 27/31 (87) | 134/158 (85) | 0.959 |
| Device implantation  Pacemaker/CRT-P  ICD/CRT-D | 25 (37)  27 (40) | 116 (41)  95 (33) | 0.610  0.281 |

Values are presented as median (interquartile range), mean ± SD, number (%), or number of positive findings per number of studied patients (%). ACE-i, angiotensin-converting enzyme inhibitor; ARB, angiotensin II receptor blocker; AVB, atrioventricular block; BNP, brain natriuretic peptide; CMRI, cardiac magnetic resonance imaging; CRT-D, cardiac resynchronization therapy-defibrillator; CRT-P, cardiac resynchronization therapy-pacing; CS, cardiac sarcoidosis; 67Ga, gallium-67; ICD, implantable cardioverter defibrillator; IQR, interquartile range; LGE, late gadolinium enhancement; LVEF, left ventricular ejection fraction; LVSWT, left ventricular septal wall thinning; NSVT, non-sustained ventricular tachycardia; SD, standard deviation; SPECT, single photon emission computed tomography; VF, ventricular fibrillation; VT, ventricular tachycardia.
