# Supplementary material for: Prognostic impact of corticosteroid maintenance dose and re-escalation in patients with cardiac sarcoidosis
Source: Open Heart. 2026 Mar 6;13(1):e004048. doi: 10.1136/openhrt-2026-004048 (PMC12970080; doi:10.1136/openhrt-2026-004048)
Supplement: online supplemental file 5 [file openhrt-13-1-s005.docx]

Supplemental Table 2 Prognostic factors for all-cause mortality in all population

|  | Univariabla | | | Multivariable | | |
| --- | --- | --- | --- | --- | --- | --- |
|  | HR | p-value | 95% CI | HR | p-value | 95%CI |
| Age (1-year increase) | 1.04 | 0.023 | 1.00–1.07 | 1.05 | 0.006 | 1.01-1.09 |
| Female sex (vs male sex) | 0.83 | 0.589 | 0.41–1.64 |  |  |  |
| Histological CS (vs clinical CS) | 0.79 | 0.528 | 0.38-1.63 |  |  |  |
| iCS (vs systemic CS) | 1.56 | 0.358 | 0.60-4.00 | 1.38 | 0.542 | 0.49-3.91 |
| AVB at CS diagnosis | 0.59 | 0.127 | 0.30–1.16 |  |  |  |
| VT at CS diagnosis | 0.68 | 0.387 | 0.28–1.62 |  |  |  |
| LVEF (1% increase) | 0.97 | 0.002 | 0.95–0.99 | 0.97 | 0.004 | 0.95-0.99 |
| Abnormal uptake of ^67^Ga scintigraphy or ^18^F-FDG PET in the heart | 3.31 | 0.266 | 0.40–27.3 |  |  |  |
| additional immunosuppressants | 1.03 | 0.969 | 0.24-4.30 |  |  |  |
| Corticosteroid re-escalation | 0.87 | 0.700 | 0.41-1.80 |  |  |  |
| Corticosteroid maintenance dose  　Recommended dose  Low dose  High dose | Reference  2.36  2.82 | 0.024  0.025 | 1.11-4.97  1.14-7.00 | Reference  2.52  3.02 | 0.018  0.031 | 1.17-5.43  1.10-8.27 |

AVB, atrioventricular block; CI, confidence interval; CS, cardiac sarcoidosis; ^18^F-FDG PET, ^18^F-fluorodeoxyglucose positron emission tomography; ^67^Ga, gallium-67; HR, hazard ratio; iCS, isolated cardiac sarcoidosis; LVEF, left ventricular ejection fraction; VT, ventricular tachycardia.
