# Supplementary material for: Prognostic impact of corticosteroid maintenance dose and re-escalation in patients with cardiac sarcoidosis
Source: Open Heart. 2026 Mar 6;13(1):e004048. doi: 10.1136/openhrt-2026-004048 (PMC12970080; doi:10.1136/openhrt-2026-004048)
Supplement: online supplemental file 6 [file openhrt-13-1-s006.docx]

Supplemental Table 3 All cause death, CV death, and VT/VF events among three maintenance dose groups

|  | All patients  (n=29) | Low-dose  (0–4.9 mg)  (n=2) | Recommended-dose  (5.0–10.0 mg)  (n=23) | High-dose  (>10.1 mg)  (n=4) |
| --- | --- | --- | --- | --- |
| All cause death | 2 | 1 (50) | 1 (4) | 0 (0) |
| CV death | 1 | 1 (50) | 0 (0) | 0 (0) |
| VT/VF events | 6 | 0 (0) | 5 (22) | 1 (25) |

CV, cardiovascular; VF, ventricular fibrillation; VT, ventricular tachycardia.
